# A Novel Spectrofluorimetric Method for Vibegron in the Newly FDA Approved Pharmaceutical Formulation and in Human Plasma: Analytical QbD Strategy for Method Development and Optimization

**DOI:** 10.1007/s10895-024-04020-0

**Published:** 2024-11-16

**Authors:** Mina Wadie, Mahmoud A. Tantawy, Zeinab M. Goda

**Affiliations:** 1 Pharmaceutical Analytical Chemistry Department, Faculty of Pharmacy, Cairo University, Kasr El-Aini Street, Cairo, ET-11562 Egypt; 2 Department of Chemistry, Faculty of Pharmacy, October 6 University, 6 of October City, Giza, ET-12573 Egypt

**Keywords:** Dansyl chloride, Human plasma, Quality-by-design, Spectrofluorimetry, Vibegron

## Abstract

Vibegron is a novel selective beta-3 adrenergic receptor agonist molecule, recently approved by US Food and Drug Administration (FDA) in tablet pharmaceutical formulation for treating overactive bladder syndrome. Such formulation necessitates the development of a simple, fast and cost-effective methodology capable of assaying the drug in various real samples with high sensitivity. Herein, a facile and robust spectrofluorimetric method was introduced, for the first time, for vibegron quantification based on analytical quality-by-design approach. The method involves drug reaction with dansyl chloride at pH 9.8, as a smart approach to overcome the non-fluorescent nature of vibegron, giving a highly fluorescent yellow derivative measured at 514 nm after being excited at 345 nm. Plausible reaction scheme between the drug and dansyl chloride was elucidated through studying the differences in their infrared (IR) spectra. Variables affecting fluorescence intensities were carefully screened and rationally optimized *via* preliminary scouting studies and central composite design for accurate and robust results. Full International Council for Harmonisation (ICH) validation protocol was followed where linearity was achieved in range of 20.0–400.0 ng/mL with minimum detectability of 3.6 ng/mL. The proposed method expressed good capability in assaying the marketed dosage forms with no excipient inference. Finally, the high sensitivity of such method paved the way for extending its application to quantify vibegron in spiked human plasma at concentrations around its real human plasma concentrations for further bioavailability studies.

**Supplementary Information:**

The online version contains supplementary material available at 10.1007/s10895-024-04020-0.

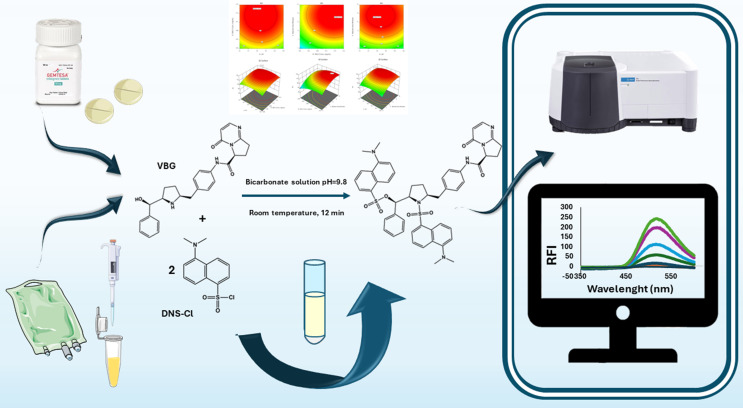

## Introduction

**Fig. 1:**
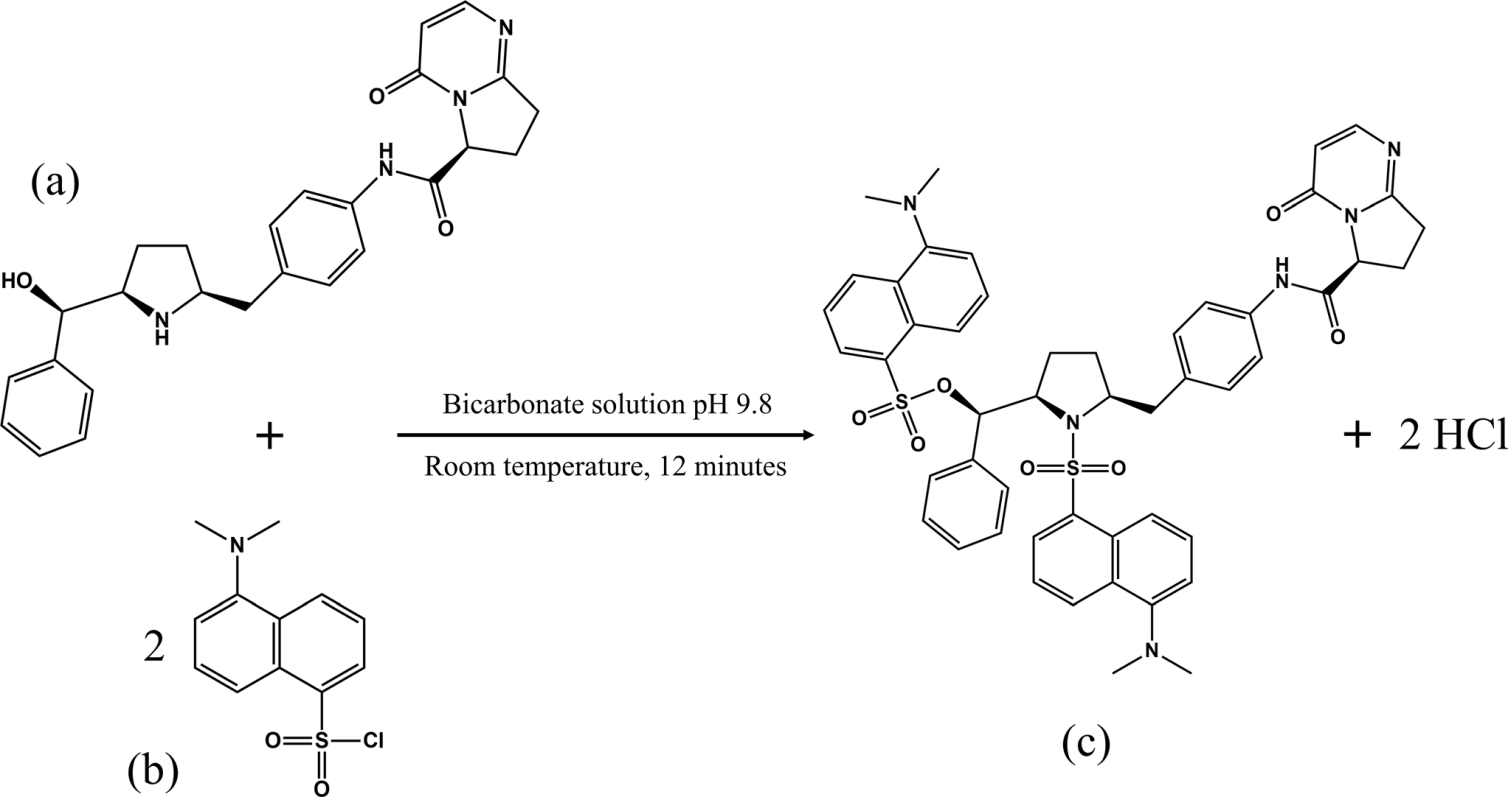
Suggested reaction scheme between (**a**) vibegron and (**b**) dansyl chloride to give (**c**) dansylated derivative

The rapid advancements in pharmaceutical sciences and healthcare pave the way to innovation of new therapeutic compounds for persistent diseases, with improved pharmacological and pharmacokinetic features. One of these novel compounds is vibegron (VBG, Fig. 1a), a selective beta-3 adrenergic receptor agonist, which has been lately permitted by the Food and Drug Administration (FDA) for treating overactive bladder syndrome [1]. It is commercially available under the brand name “Gemtesa^®^” tablets of dosage strength of 75 mg with maximum plasma concentration (C_max_) ranging from 80 to 110 ng/mL [2]. Daily administration aids in relaxing the detrusor bladder muscle and inhibition of its hyperactivity thus increasing retention of urine with less micturition frequency [3, 4]. One more promising aspect of this formulation is being swallowed as intact or crashed, hence improving medication adherence of elderly patients suffered with dysphagia and swallowing difficulties [2]. In order to comprehensively quantify VBG in quality control laboratories or sensitivity assessing their plasma concentrations, the literature lacked any reliable analytical methods for VBG determination except a reported LC-MS/MS one in human plasma only [5]. These findings have acted as the impetus for our endeavor to develop a simple, fast and cost-effective methodology capable of assaying VBG in various real samples with high sensitivity.

In the realm of analytical spectroscopy, spectrofluorimetry provides a promising candidate for pharmaceutical analysis owing to its simplicity, ease-of-use, high sensitivity and time effectiveness [6–9]. It also sticks to the principles of green analytical chemistry through utilizing less solvents, low energy (less than 0.1 kWh per sample) as well as limited waste generation compared to other chromatographic methods [10–16]. All these merits boost the spectrofluorimetric technique as an economic and reliable analytical platform, particularly in modest quality control laboratories, for determining drugs in various matrices [17–19]. However, the development of spectrofluorimetric methods engage numerous experimental parameters that demands an in-depth understanding and careful optimization. To solve these limitations, multivariate experimental designs are introduced for rapid screening method variables followed by response surface analysis for optimizing critical ones [20–22]. These systematic procedures are considered the key steps for “Analytical Quality-by-Design (AQbD)” approach that aims to build quality and robustness during method development, rather than their monitoring in the final stage. Thus, AQbD approach paves the way for excluding revalidation procedures upon method transfer among laboratories which in turn saves time and resources [23–25].

The main perquisite for conducting fluorometric analysis is possessing the analyte native fluorescent emission however, several pharmaceutical compounds are non-fluorescent. For this reason, researchers’ attention was directed to induce fluorescence for non-fluorescent compounds *via* different chemical and physical treatments such as derivatization reactions with fluorescent probes. A wide array of fluorescent probes is reported to react specifically with several functional groups [26]. Among them is dansyl chloride (DNS-Cl, Fig. 1b) which is chemically known as 5-(dimethylamino) naphthalene-1-sulfonyl chloride [27]. It is well known to react through nucleophilic substitution with compounds having active hydrogen-functional groups such as primary & secondary amines and alcoholic hydroxyl groups [26, 28]. As a result, DNS-Cl has been widely reported as a fluorogenic reagent for the analysis of various pharmaceutical drugs, producing highly fluorescent products [29, 30]. On reviewing literature, all the reported spectrofluorimetric methods, based on DNS-Cl derivatization, were optimized *via* one variable at a time approach which is tedious and time consuming [10, 30–33]. No work has adopted yet AQbD multivariate approach for variables optimization and studying their interaction effects.

Based on the previous regards, this work is dedicated to developing, for the first time, a facile, cost-effective and highly sensitive analytical method for quantification of VBG with eschewing highly-cost analytical techniques. To achieve this goal, a robust spectrofluorimetric method was proposed in consideration with the quality principles of AQbD approach and experimental design for method optimization. The method was based on solving the non-fluorescent nature of VBG *via* its direct derivatization with DNS-Cl to get highly fluorescent yellow product. Furthermore, IR characterization was conducted to give a possible framework for the reaction between the drug and DNS-Cl. The suggested method was applied for drug investigation in the newly FDA approved formulation with high selectivity in presence of tablet excipients. Finally, the work made good use of ultra-sensitive capability of the proposed technique for drug assaying in spiked human plasma samples at concentrations around its real human plasma concentrations (C_max_).

## Experimental

### Chemicals and Reagents

Reagents, analytical grade chemicals, and bi-distilled water were used. DNS-Cl was acquired from Sigma-Aldrich (Germany) and a stock solution of 0.15 mg/mL DNS-Cl was created by dissolving 15 mg into 100 mL of acetone. Sodium bicarbonate, sodium hydroxide, 1,2-dichloromethane, acetone, chloroform, n-hexane, diethyl ether, methanol, ethyl acetate and 1-butanol were acquired from El-Nasr Co. (Egypt). A 0.5 M aqueous solution of sodium bicarbonate was prepared using water, and the pH was adjusted to 9.8 using a 0.1 M solution of sodium hydroxide with the assistance of a pH meter. The solution was stored in the refrigerator and remained viable for approximately one week. The study procured fresh human plasma samples from VACSERA, a holding business specializing in biological products and vaccinations, located in Giza, Egypt. The plasma samples were stored at freezing temperatures until they were analyzed, following a careful thawing process.

### Instruments and Software

A Cary Eclipse Fluorescence Spectrophotometer, manufactured by Agilent Technologies, USA, was utilized in conjunction with the Cary WinFLR software to conduct spectrofluorimetric measurements. The measurements were performed at a scan rate of 600 nm/min, with a slit width of 5.0 nm. The measurements were conducted in a quartz cell with a length of 1 cm at ambient room temperature. A Jenway 3505 pH meter from Staffordshire, UK was utilized to make pH adjustments. The IR spectrophotometric measurements were conducted using a Shimadzu IR Spectrophotometer (Model 435, Kyoto, Japan). Designs for experimentation during the screening and optimization stages were created using Design-Expert^®^ software (Stat-Ease Inc., Minneapolis-USA).

### Samples

Pure VBG (≥ 98%) was acquired from Ami Lifesciences PVT LTD, located in Karakhadi – India. The Gemtesa^®^ tablets, manufactured by Sumitomo Pharma Co., Ltd. in the USA, contain 75.0 mg of the active pharmaceutical ingredient per tablet. The Gemtesa^®^ tablets with batch number 08725 were purchased from the USA market.

### Standard Solutions

Methanol was the chosen solvent for VBG [5] where stock solution (50.0 µg/mL) was prepared by transferring a precisely weight 5.0 mg of the pure VBG to a 100 mL volumetric flask and dissolved in it. The volume was filled up to the designated level using the same solvent. An aliquot of the stock solution was diluted with methanol to a final volume of 100 mL in order to achieve the necessary working concentration.

### Procedures

#### Derivatization Step

Various volumes of the working drug solution, ranging from 20.0 to 400.0 ng/mL, were placed in 10 mL stoppered glass tubes. Then, 0.3 mL of 0.5 M sodium bicarbonate solution with a pH of 9.8 and 0.3 mL of a DNS-Cl solution with a concentration of 0.15 mg/mL were added to each tube. The resulting solution was mixed vigorously through by a vortex mean. The tubes were allowed to equilibrate at room temperature for a duration of 12.0 min. The fluorescent compound was isolated using 5 mL of methylene chloride, and the fluorescence of the resulting organic layer was measured at an emission wavelength of 514 nm after being excited at 345 nm. The fluorescence correction was obtained by applying the same process to a blank reagent and subtracting its observed fluorescence intensity.

#### Construction of Calibration Curves

A standard calibration with concentrations ranging from 20.0 to 400.0 ng/mL of VBG was prepared using the previously described procedures under: ***Derivatization step***. To obtain the relative fluorescence intensity (RFI), the resulting spectrum of the blank experiment was subtracted from each spectrum. Calibration graphs were created by graphing concentrations in ng/mL against RFI at 514 nm. Subsequently, the regression equations that corresponded to the data were computed.

#### Procedures for Commercial Tablets

Exactly ten tablets of the marketed Gemtesa^®^ dosage form were precisely weighed, crushed, and mixed thoroughly to create a uniform mixture. A precise weight equivalent to 5.0 mg of the powdered tablets was subjected to sonication with an appropriate volume of menthol in a 100 mL calibrated flask for a duration of 20 min. Once the solution was diluted with methanol up to the designated mark, it was then filtered. Once the filtrate was diluted to provide solution with the desired concentration, the suggested analytical method was utilized.

#### Procedures for Spiked Human Plasma

Human plasma free from drugs was held at a temperature of -20 °C until it was ready for analysis. Prior to usage, it was thawed to room temperature. A 450.0 µL portion of human plasma was transferred into multiple centrifuge tubes and spiked with 50 µL VBG working standard solutions to achieve a final concentration range of 20.0–250.0 ng/mL. The precipitation of plasma proteins was achieved by using 3.0 mL of acetonitrile. The mixture was vortexed for 2 min and then subjected to centrifugation at 4000 rpm for 20 min. The supernatant obtained was dried completely using a nitrogen gas stream. The remaining mass was restored using methanol and thereafter analyzed following the same procedure described in the “***Derivatization step***”. A blank value was ascertained by handling the plasma sample without the drug in a similar manner.

## Results and Discussion

This work aimed to make good use of spectrofluorimetry, as being highly sensitive, rapid and cost-effective technique, for determination of VBG in various real samples. To achieve this goal and upon reviewing chemical structure of the studied drug, it is characterized with two active hydrogens at aliphatic alcoholic and cyclic pyrrolidine functional groups. This paved the way for its conversion from non-fluorescent compound to highly fluorescent yellow product *via* simple derivatization step with a fluorogenic probe like DNS-Cl [27]. The fluorescent product was extracted with organic solvent then measured at 514 nm after being excited at 345 nm as shown in Figure S1.

Concerning extraction procedure, it is attributed to the co-existence of a secondary highly fluorescent and polar product, entitled “dansyl hydroxide”, in the aqueous reaction medium along with the main derivatization product. This dansyl hydroxide was formed upon the addition of DNS-Cl in the alkaline medium and would express high interference upon direct measurements. Such limitation was solved by an extraction procedure with an immiscible organic solvent prior to fluorometric measurements. Consequently, the polar dansyl hydroxide settled in the aqueous phase while the main derivatized product extracted to immiscible organic layer [30, 34].

### Analytical Quality-by-Design for Method Development and Optimization

Studying various method variables, in-depth understanding their interactions as well as careful optimization were performed as per a systematic AQbD framework which involves the following main steps [35–37].

#### Defining the Analytical Target Profile and Attributes

The initial stage in establishing an analytical technique involves clearly defining the analytical target profile (ATP), which outlines the primary objectives and anticipated results of the method [21]. Herein, our ATP was the development of highly sensitive, uncomplicated and rapid analysis method for VBG in worldwide quality control laboratories, with excluding traditional & costly chromatographic techniques. Consequently, spectrofluorimetric procedures were adopted where achieving high relative fluorescence intensity (RFI) was set to the main critical analytical attributes (CAA) that measured and gave indication about method performance.

#### Quality Risk Assessment and Scouting Studies

**Fig. 2:**
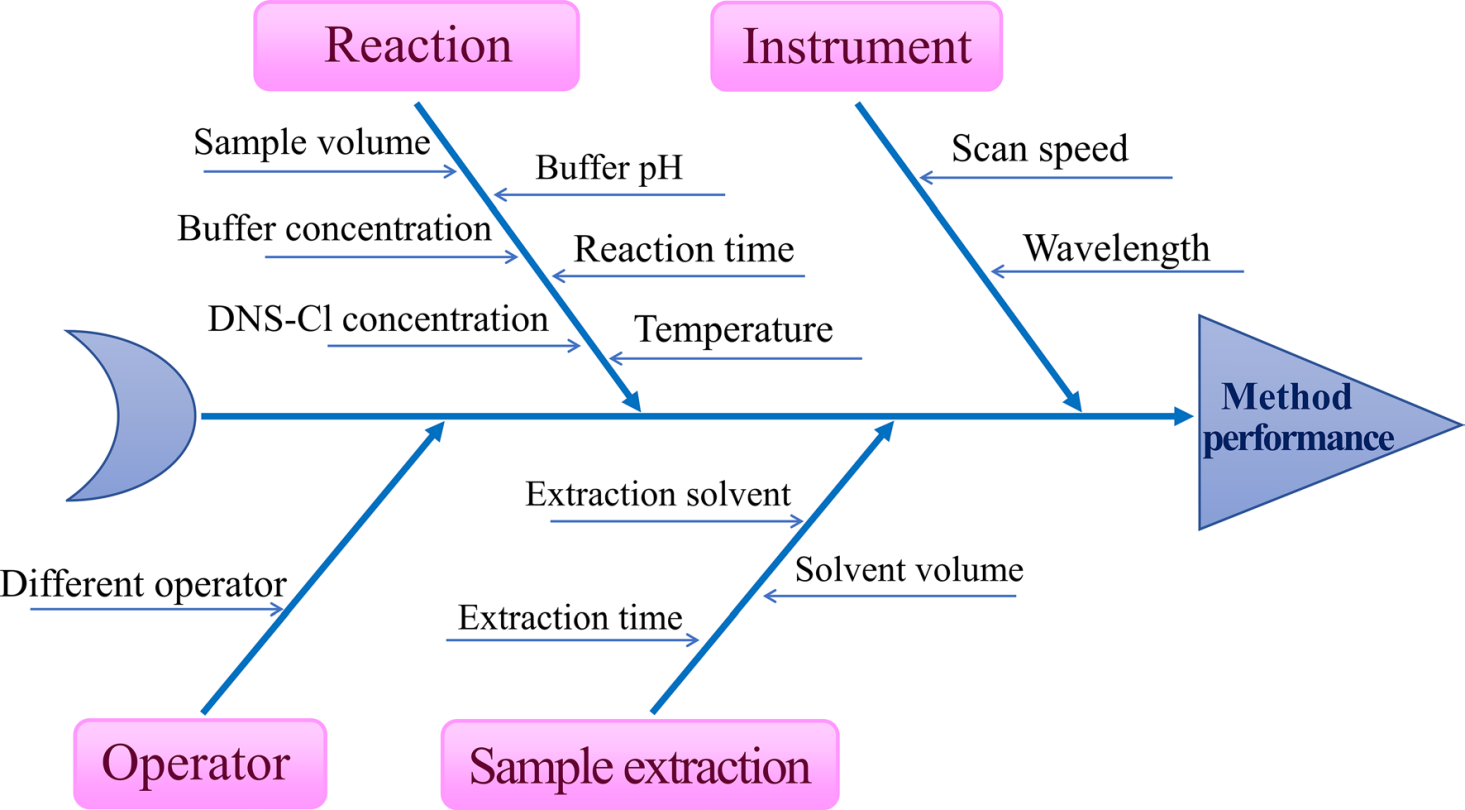
Proposed Ishikawa fishbone diagram for the proposed spectrofluorimetric method

Establishment of spectrofluorimetric method based on DNS-Cl derivatization is a multi-parameter process, and thus requiring studying and monitoring. First of all, a science-based benchmark termed “quality risk assessment” was conducted as a brainstorming protocol to identify various risk factors that may pose a risk to method quality [35, 38]. Herein, Ishikawa fishbone diagram was utilized as quality risk assessment tool to facilitate subsequent screening and optimization processes for the studied spectrofluorimetric method as illustrated in Fig. 2. This was followed by performing serval preliminary experiments to study the impact of each factor in addition to identifying the critical method parameters (CMP) and their levels.

Based on previous literature, reaction of DNS-Cl is proceeded under alkaline conditions with pH values ranging from 9 to 10.5 where RFI was practically found to be influenced [33, 39]. Consequently, working pH value was set as the first CMP that required fine optimization with statistical analysis. Moreover, scouting studies during method development found that DNS-Cl concentration and reaction time remarkably influenced RFI of the obtained product thus further optimization studies were demanded. On the other hand, the influence of varying buffer concentration, applied sample volume and reaction temperature was insignificant on RFI upon varying in range of 0.3–0.7 M, 20–100 µL and 25–35 °C, respectively, Figures S2a-c. Thus, procedures were followed using 0.5 M bicarbonate buffer at room temperature (25 °C) with a sample volume (50 µL).

Regarding extraction procedures, multiple organic solvents, immiscible with water, were tried including ethyl acetate, diethyl ether, dichloromethane (methylene chloride) and chloroform. Upon measurements, ethyl acetate gave high fluorescence values in blank samples and was excluded. However, dichloromethane gave the highest RFI among other solvents with less toxicity, making it the selected one for our work, Table 1. Time of extraction is another factor controlling extraction process where different time intervals (3–15 min) were monitored with continuous vortex. It was found that maximum extraction was attained after 10 min and remain stable after that as illustrated in Figure S2d.

**Table 1:** The fluorescence intensities of vibegron dansylated derivatives along with their maximum excitation (λ_ex_) & emission (λ_em_) wavelengths in various organic extraction solvents

Extraction solvent	λex / λem (nm)	Fluorescence intensity	
		Sample ^*^	Blank
Ethyl acetate	332 / 502 nm	350.48	230.36
Diethyl ether	336 / 508 nm	100.35	2.16
Dichloromethane	345 / 514 nm	118.07	2.62
Chloroform	342 / 512 nm	107.25	2.34

^*^ Vibegron concentration of 200 ng/mL

#### Experimental Design for Method Optimization

Based on the preliminary experiments and several scouting studies, working pH, DNS-Cl concentration and reaction time were found to significantly impact method outcomes and were set as our CMPs. Response surface methodology was conducted based on face central composite design to explore the most favorable adjustment for these CMPs. Randomized twenty experiments were designed and performed involving six center points for assuring method accuracy where the obtained RFI values were recorded, Table S1. As declared in the table, working pH was investigated along its reported range at three levels (9.5, 10.0 & 10.5) with adding 0.3 mL of DNS-Cl solution of different concentrations (0.05, 0.1 & 0.15 mg/mL). Concerning reaction time, the reaction was monitored after three-time interval levels (5, 10 & 15 min). After that, the created model was analyzed where second-order polynomial equation was the fit one considering linear, factors interactions and quadratic effects, Table 2. ANOVA and fit statistics of the regression model were revised to assure model validity for prediction. Satisfactory R^2^ values and non-significant lack-of-fit are summarized in Table 2 reflected by random scattering of residuals and normal distribution of residual diagnostic plots [40, 41], Figure S3.

**Table 2:** Analysis of variance (ANOVA) results with coefficients terms of their second-order polynomial equations and fit statistics for face central composite optimization design

Coefficient terms	Estimate	*p*-value ^a^
A-pH	-0.382	0.0007
B-DNS-Cl Conc	5.2	< 0.0001
C-Reaction time	7.31	< 0.0001
AB	0.06	0.5102
AC	-0.1175	0.2108
BC	-0.215	0.0344
A²	-4.81	< 0.0001
B²	-4.86	< 0.0001
C²	-6.96	< 0.0001
*ANOVA and fit statistics*	*Relative fluorescence intensity (RFI)*	
p-value for model ^a^	< 0.0001	
p-value for Lack of fit ^a^	0.1549	
R^2 b^	0.9997	
Adjusted R^2 b^	0.9994	
Predicted R^2 b^	0.9983	

^a^ Significantly different at *p* ≤ 0.05

^b^ Values above 0.8 are acceptable

**Fig. 3:**
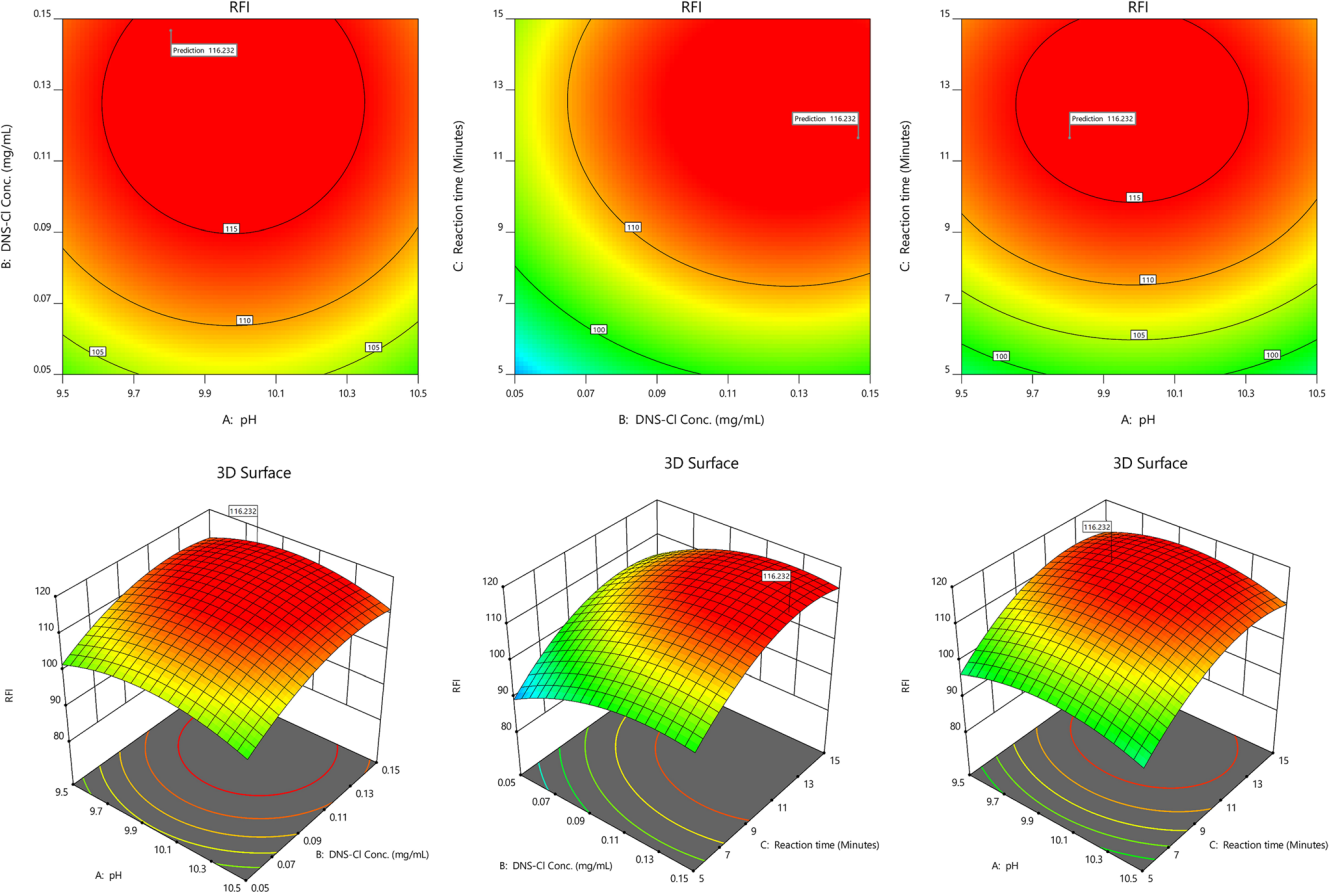
Contour surface and 3D surface plots showing the effects of working pH, DNS-Cl concentration and reaction time on relative fluorescence intensity (RFI)

Multi-response optimization tool was then utilized to find the CMPs levels that achieve maximum RFI and the highest Derringer’s desirability value. The tool was set with the following goals: working pH & DNS-Cl concentration “In range”, reaction time “Minimize” while the RFI set at “Maximize”. As declared by contour surface plots and 3D response surfaces graphs in Fig. 3, the predicted optimum conditions were working pH of 9.8 and DNS-Cl solution with concentration of 0.15 mg/mL where reaction time of 12.0 min was enough to achieve highest RFI.

### IR Characterization and Reaction Scheme Elucidation

Referring to the chemical structure of our studied drug (Fig. 1a), VBG possesses two functional group moieties with active hydrogen, *namely*; aliphatic alcoholic hydroxyl group and secondary amine [27]. These are supposed to make nucleophilic attack on the sulfonyl chloride groups of DNS-Cl giving a highly fluorescent yellow derivative as illustrated by chemical scheme in Fig. 1. To verify such plausible reaction scheme, IR characterizations were performed for the derivatization product as well as VBG drug and DNS-Cl using potassium bromide (KBr) discs. The derivatization product was prepared under the settled optimum conditions, extracted by dichloromethane and then left to dry under room temperature. As shown in Figure S4, the IR spectrum of VBG is characterized with the existence of N-H stretching ones of secondary amine & amide of medium intensity and strong O-H stretching bands of aliphatic alcoholic group at 3317 and 3275 cm^− 1^, respectively [42]. In addition, other IR bands were displayed at 2827 cm^− 1^ for aliphatic C-H, 1722 cm^− 1^ for aromatic C-H and 1666 cm^− 1^ for C = O in ketone and amidic groups. On the other hand, DNS-Cl is characterized with stretching bands of strong intensity for S꞊O moiety in sulfonyl chloride group at 1207–1145 cm^− 1^, Figure S5. Upon reaction, two main differences than VBG’s IR spectrum are observed: the disappearance of O-H stretching bands at 3275 cm^− 1^ as well as the appearance of strong bands 1207–1145 cm^− 1^ for S꞊O moiety in sulfonyl chloride group [42]. This is accompanied by slight decreasing the intensity of N-H bands at 3317 cm^− 1^ without its disappearance. This may be attributed to N-H moiety of amide group is barely available for nucleophilic substitution and not participate in the reaction, Figure S6.

### Analytical Method Validation

The suggested method was verified in accordance with the International Council for Harmonisation (ICH) Q2 (R1) recommendations [43] for range, linearity, limit of detection (LOD), limit of quantification (LOQ), robustness, accuracy, and precision.

#### Linearity and Sensitivity

**Fig. 4:**
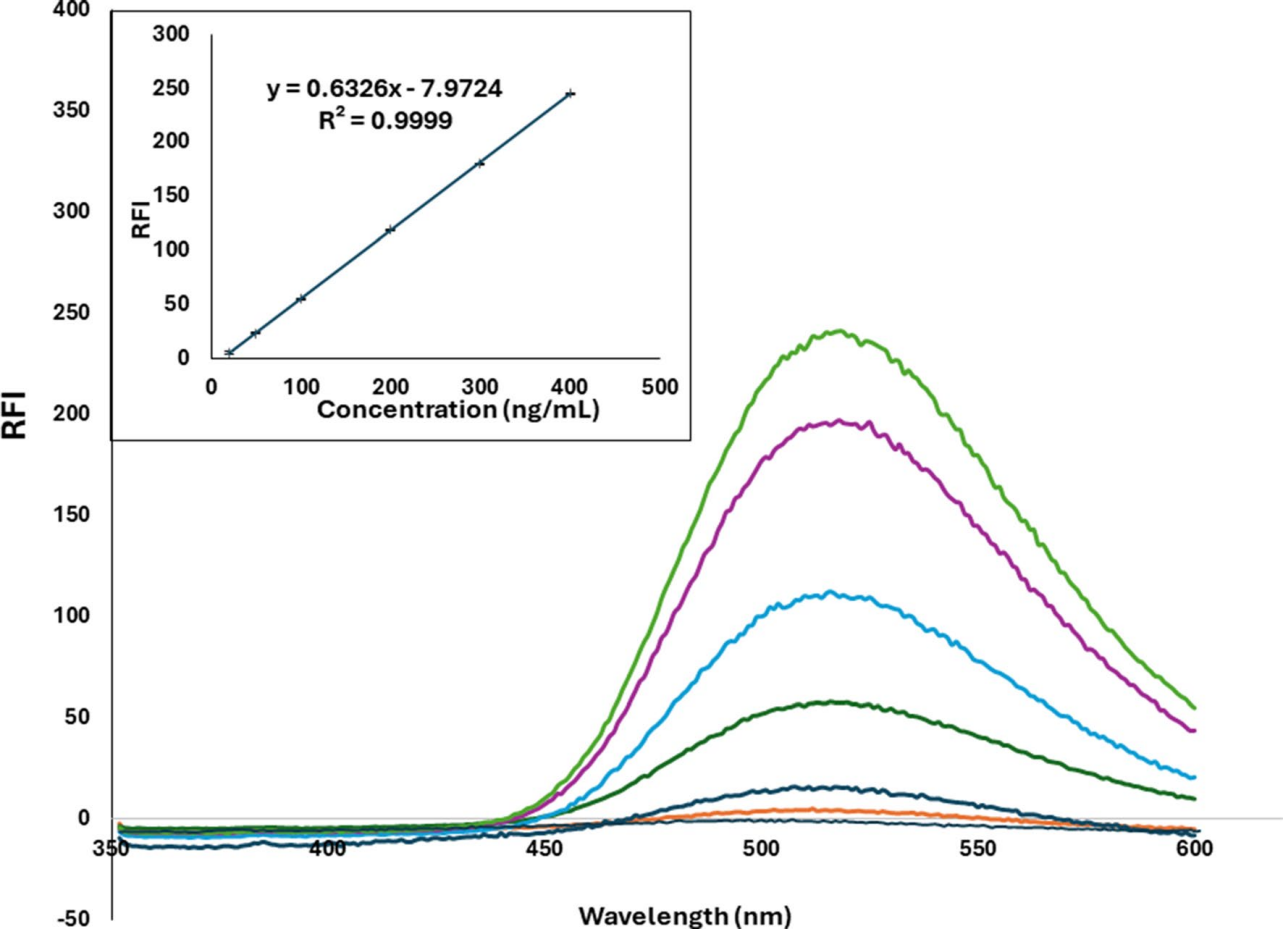
Linearity of RFI at 514 nm to the corresponding concentrations of vibegron (20.0– 400.0 ng/mL)

To assess linearity at the specified conditions, the VBG was determined at six different concentration levels (*n* = 6) for the standard solution. The calibration curve of VBG exhibited linearity within the concentration range of 20.0–400.0 ng/mL VBG, as depicted in Fig. 4. The data acquired, as shown in Table 3, demonstrated the strong linearity of the suggested method. LOD and LOQ were determined using the formulas 3.3 σ/S and 10 σ/S, respectively. Here, σ represents the standard deviation of the residuals of the regression line, and S is the slope of the regression line. The proposed spectrofluorimetric method had a LOD of 3.6 ng/mL and a LOQ of 11.0 ng/mL for VBG. The analytical performance parameters of the proposed approach are outlined in Table 3.

**Table 3:** Regression and validation parameters for the determination of vibegron by the proposed spectrofluorimetric method

Parameter	Vibegron
Range (ng/mL)	20.0–400.0
Slope	0.6326
Intercept	− 7.9724
SD of Slope	0.006
SD of Intercept	1.278
SD of Residuals	0.695
Correlation coefficient (r)	0.9999
Accuracy ^a^ (Mean recovery % ± SD)	99.29 ± 0.880
LOD (ng/mL)	3.6
LOQ (ng/mL)	11.0
Robustness ^b^	1.129
Repeatability (intraday) ^c^	0.569
Intermediate precision (interday) ^d^	0.983

^a^ Average of three determinations (40.0, 150.0 and 350.0 ng /mL VBG)

^b^ Robustness: pooled % RSD (average of three concentrations of three replicate each (*n* = 9) analyzed in different conditions

^c^ The intraday (*n* = 3), % RSD of determination of three concentrations of VBG (50.0, 200.0 and 300.0 ng/mL) repeated three times within the day

^d^ The interday (*n* = 3), % RSD of determination of three concentrations of VBG (50.0, 200.0 and 300.0 ng/mL) repeated three times in three days

#### Accuracy

The accuracy of the recommended approach was evaluated by measuring the % recovery of three different VBG concentrations. Each experiment was conducted three times. The results are displayed as the mean and standard deviation of the percentage recovery, as presented in Table 3. The close proximity of the % recovery values to 100% at all the concentrations examined indicated the satisfactory level of accuracy achieved by the current method.

#### Precision

To assess interday and intraday precision, the suggested approach was used to investigate three different concentrations within the linearity range. Intraday precision was evaluated within the same day, while interday precision was evaluated over three consecutive days. The trials were conducted three times each. The effectiveness of the proposed method was demonstrated by the low computed % RSD value, as indicated in Table 3.

#### Robustness

The robustness of the proposed analytical methodology was evaluated by introducing minor changes in the experimental circumstances, such as adjusting the excitation and emission wavelengths within a range of ± 2.0 nm, varying the pH by ± 0.1, and modifying the volume of DNS-Cl by ± 0.1. The findings in Table 3 demonstrated that a slight alteration in these parameters did not have a substantial impact on the method’s performance, as evidenced by a % RSD of less than 2.0%.

### Applications of the Suggested Spectrofluorimetric Method

#### Assay of the Marketed Gemtesa Tablets

The spectrofluorimetric method suggested in this study was successfully used to analyze VBG in its tablet dosage form. A high value of % recovery was obtained (99.56 ± 0.878). In addition, the substantial percentage of recovery gave proof of the lack of major interference from typical excipients, as demonstrated in Table 4.

**Table 4:** Application of the proposed method for determination of VBG in Gemtesa ^®^ tablets and spiked human plasma

Application	Gemtesa^®^ tablets (75 mg/tablet)		Spiked human plasma	
	Found% ± RSD ^a^		Nominal concentration (ng/mL)	Recovery% ± CV ^b^
Vibegron	99.56 ± 0.878		20.0	107.98 ± 3.840
			50.0	97.51 ± 1.429
			100.0	99.77 ± 2.286
			150.0	99.72 ± 2.272
			200.0	99.55 ± 1.830
			250.0	100.46 ± 0.780

^a^ Results are the average value of three determinations

^b^ Six replicates per concentration level

#### Application to Spiked Human Plasma

To assess linearity under the specified conditions, we conducted a VBG determination on plasma samples at six different concentration levels (*n* = 6) [29, 33, 44, 45]. The calibration curves of VBG exhibited a good linearity within the concentration range of 20.0–250.0 ng/mL. The data shown in Table 4 indicate that the coefficient of variation (CV) and percent average of recovery for the prepared spiked plasma samples fell within the range of 0.780–3.840% and 97.51–107.98%, respectively. To assure results validity of the proposed spectrofluorimetric methods, five selected concentration levels in spiked human plasma (*n* = 5) was analyzed by our method and by the reported LC-MS/MS one [5]. Statistical analysis was then performed at p-value of 0.05 using Student t-test and F-test where the computed values were found to be less than theoretical ones, Table S2. Such observation disclosed the absence of any significant difference between the proposed and official method in terms of analytical performance. Therefore, it is evident that the proposed method is appropriate for analyzing the investigated medication in human plasma.

## Conclusion

The suggested spectrofluorimetric technique presents numerous advantages. Firstly, the process of derivatization leads to a substantial enhancement in the fluorescence signal, hence enhancing the sensitivity of the method. Furthermore, the dansyl chloride derivative exhibits a significant difference between its excitation and emission wavelengths (Stokes shift), which serves to reduce any potential interference from excipients, hence improving the method’s specificity. The approach was implemented to determine the examined drug in its pure form, pharmaceutical tablets formulation, and spiked human plasma. The method was able to accurately measure the drug without any interference from common excipients or other components that may be present in denatured plasma. Additionally, the method had a minimal detection limit of 3.6 ng/mL. Despite the relatively higher sensitivity acquired by the only reported LC-MS/MS method, our proposed one is the first to determine VBG in the marketed pharmaceutical tablets. Moreover, the proposed technique provides a simpler and faster approach for preparing and analyzing samples, making it well-suited for regular quality control purposes and clinical laboratories.

## Electronic Supplementary Material

Below is the link to the electronic supplementary material.

Supplementary Material 1

## Data Availability

No datasets were generated or analysed during the current study.
