# Supplementary material for: A Novel Spectrofluorimetric Method for Vibegron in the Newly FDA Approved Pharmaceutical Formulation and in Human Plasma: Analytical QbD Strategy for Method Development and Optimization
Source: J Fluoresc. 2024 Nov 16;35(8):6653–63. doi: 10.1007/s10895-024-04020-0 (PMC12476424; doi:10.1007/s10895-024-04020-0)
Supplement: Supplementary file 1 — Supplementary Material 1 [file 10895_2024_4020_MOESM1_ESM.docx]

Supplementary material

for

**A novel spectrofluorimetric method for vibegron in the newly FDA approved pharmaceutical formulation and in human plasma: Analytical QbD strategy for method development and optimization**

***Mina Wadie ^a,^*, Mahmoud A. Tantawy ^a,b^, Zeinab M. Goda ^a^***

*^a^ Pharmaceutical Analytical Chemistry Department, Faculty of Pharmacy - Cairo University, Kasr El-Aini Street, ET-11562, Cairo - Egypt*

*^b^ Department of Chemistry, Faculty of Pharmacy, October 6 University, 6 of October City, Giza, Egypt*

*Corresponding author. Tel.: +20 1277978958

e-mail address: mina.wa.yousif@pharma.cu.edu.eg

**Figure S1**. Excitation and emission spectra of 400 ng/mL vibegron - dansyl chloride reaction product (1 and 2) against non-derivatized drug (3 and 4).


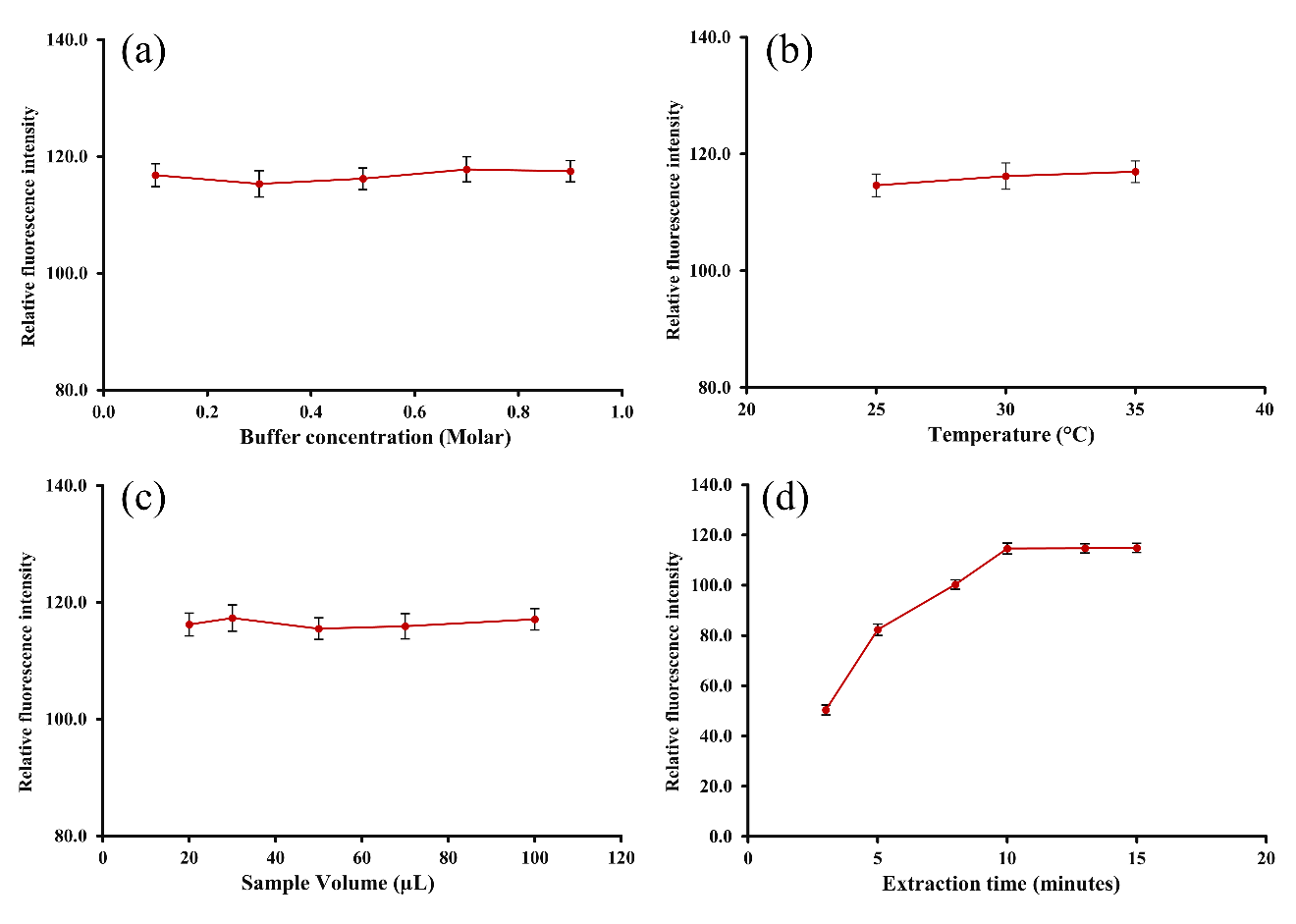


**Figure S2.** Scouting studies for various parameter affecting the proposed spectrofluorimetric method for vibegron determination.

**
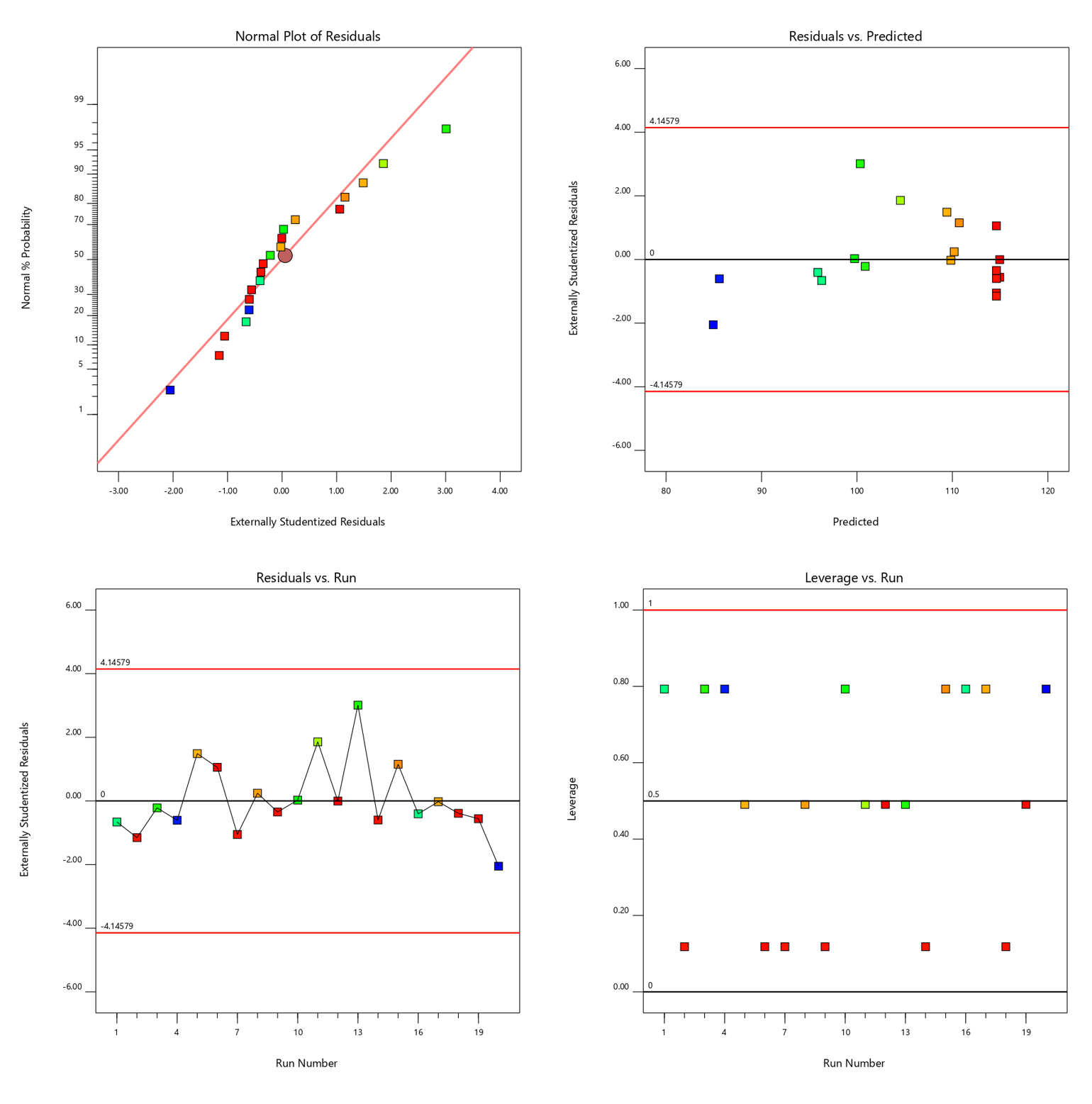
**

**Figure S3.** Plots of normal probability and residuals for relative fluorescence intensity (RFI) model using face central composite design.


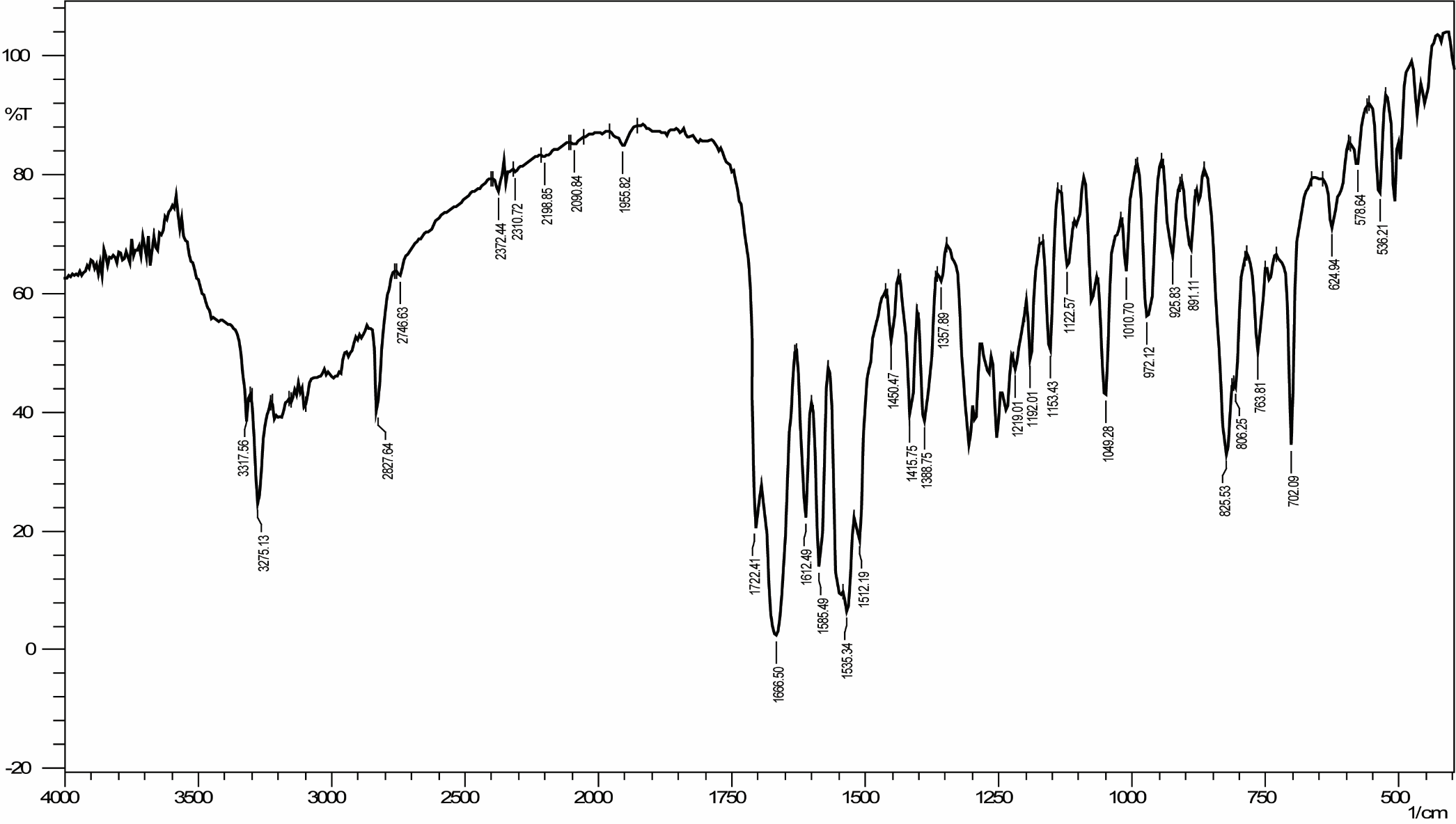


**Figure S4.** Infrared spectrum of vibegron.


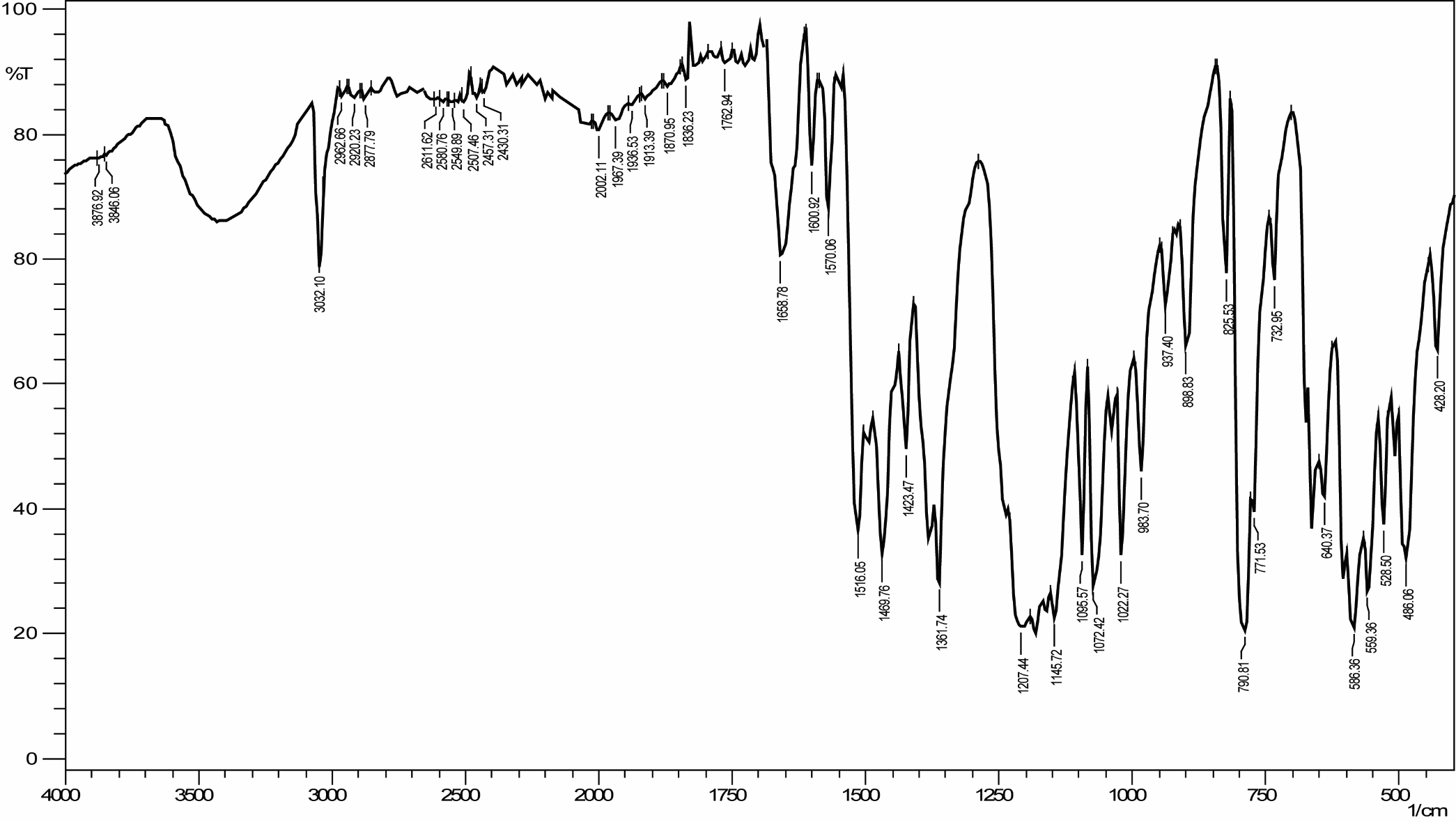


**Figure S5.** Infrared spectrum of dansyl chloride.


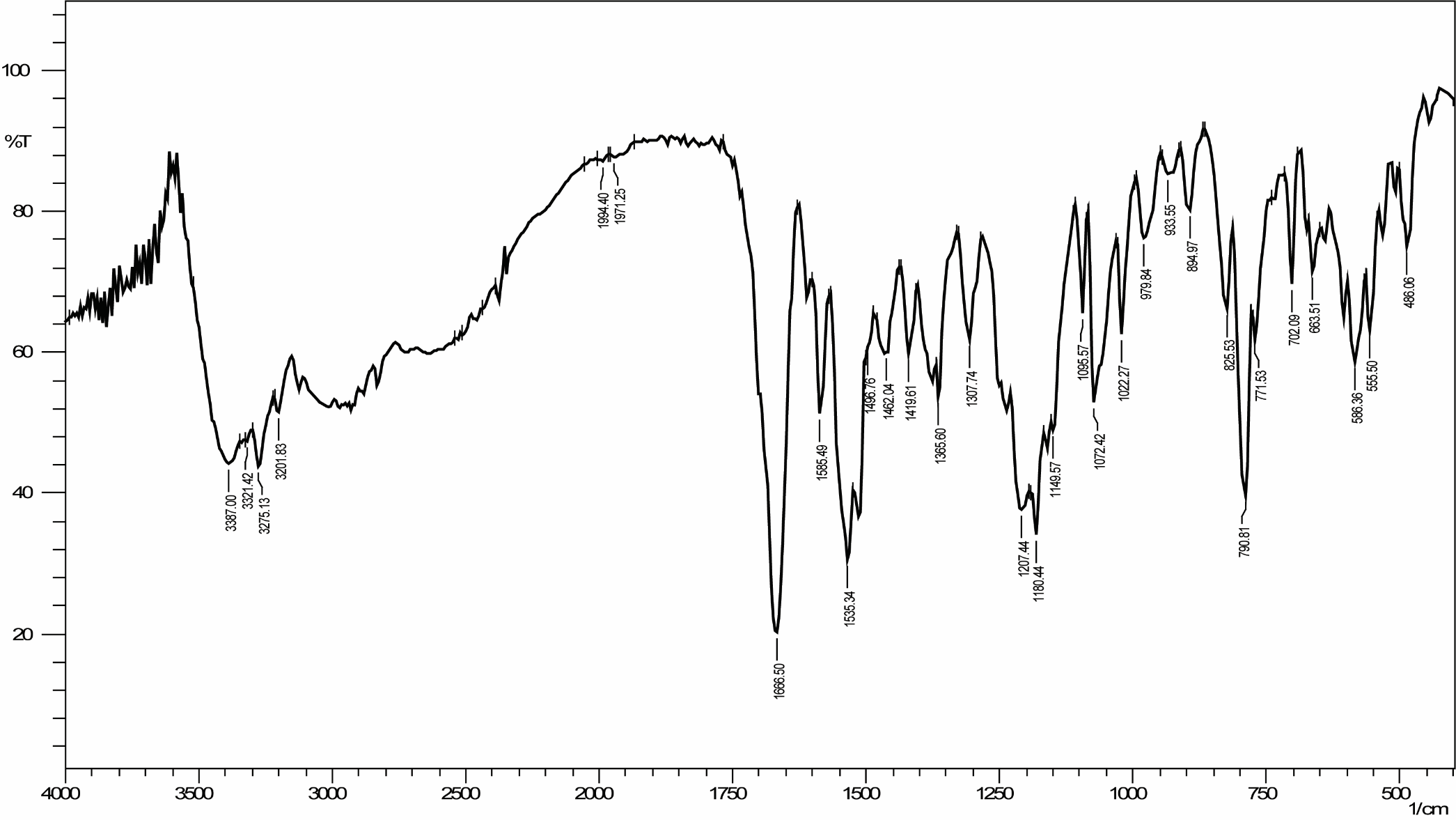


**Figure S6.** Infrared spectrum of derivatization of vibegron with dansyl chloride.

**Table S1.** Face centered composite design using three-level three factors in their actual values along
 with the recorded response for method optimization.

| Number of experiments | Design factors | | |  | Design responses |
| --- | --- | --- | --- | --- | --- |
|  | Actual Values | | |  | Relative Fluorescence Intensity (RFI) ^*^ |
|  | Working pH | DNS-Cl concentration (mg/mL) | Rection time (minutes) |  |  |
| 1 | 9.5 | 0.15 | 5 |  | 96.23 |
| 2 | 10 | 0.1 | 10 |  | 114.35 |
| 3 | 9.5 | 0.05 | 15 |  | 100.84 |
| 4 | 9.5 | 0.05 | 5 |  | 85.52 |
| 5 | 10.5 | 0.1 | 10 |  | 109.67 |
| 6 | 10 | 0.1 | 10 |  | 114.86 |
| 7 | 10 | 0.1 | 10 |  | 114.37 |
| 8 | 9.5 | 0.1 | 10 |  | 110.23 |
| 9 | 10 | 0.1 | 10 |  | 114.53 |
| 10 | 10.5 | 0.05 | 15 |  | 99.75 |
| 11 | 10 | 0.05 | 10 |  | 104.85 |
| 12 | 10 | 0.15 | 10 |  | 114.96 |
| 13 | 10 | 0.1 | 5 |  | 100.75 |
| 14 | 10 | 0.1 | 10 |  | 114.47 |
| 15 | 9.5 | 0.15 | 15 |  | 110.85 |
| 16 | 10.5 | 0.15 | 5 |  | 95.85 |
| 17 | 10.5 | 0.15 | 15 |  | 109.84 |
| 18 | 10 | 0.1 | 10 |  | 114.52 |
| 19 | 10 | 0.1 | 15 |  | 114.86 |
| 20 | 10.5 | 0.05 | 5 |  | 84.74 |

^*^ Vibegron concentration of 200 ng/mL.

**Table S2.** Statistical comparison of the results obtained by the proposed Spectrofluorimetric method and the reported LC-MS/MS one for the analysis of vibegron in spiked human plasma.

| Parameter | Proposed Spectrofluorimetric method |  | Reported LC-MS/MS method |
| --- | --- | --- | --- |
| Mean of recoveries | 100.91 |  | 99.93 |
| S.D. | 4.065 |  | 3.775 |
| Variance | 16.528 |  | 14.249 |
| n | 5 |  | 5 |
| Student’s t-test (2.306)^*^ | 1.860 |  | - |
| F-test (6.388)^*^ | 1.160 |  | - |

^*^ The values in parentheses are the corresponding tabulated values at P ꞊ 0.05.
